# Cigarette smoke promotes oral leukoplakia via regulating glutamine metabolism and M2 polarization of macrophage

**DOI:** 10.1038/s41368-021-00128-2

**Published:** 2021-08-09

**Authors:** Yanan Zhu, Shuo Zhang, Jiahui Sun, Tingting Wang, Qin Liu, Guanxi Wu, Yajie Qian, Weidong Yang, Yong Wang, Wenmei Wang

**Affiliations:** 1 Nanjing Stomatological Hospital, Medical School of Nanjing University, Nanjing, China; 2 State Key Laboratory of Analytical Chemistry for Life Science & Jiangsu Key Laboratory of Molecular Medicine, Medical School, Nanjing University, Nanjing, China; 3 The State Key Laboratory of Pharmaceutical Biotechnology, Division of Immunology & Jiangsu Key Laboratory of Molecular Medicine, Medical School, Nanjing University, Nanjing, China

**Keywords:** Mechanisms of disease, Mucositis

## Abstract

Oral immunosuppression caused by smoking creates a microenvironment to promote the occurrence and development of oral mucosa precancerous lesions. This study aimed to investigate the role of metabolism and macrophage polarization in cigarette-promoting oral leukoplakia. The effects of cigarette smoke extract (CSE) on macrophage polarization and metabolism were studied in vivo and in vitro. The polarity of macrophages was detected by flow cytometric analysis and qPCR. Liquid chromatography-mass spectrometry (LC-MS) was used to perform a metabolomic analysis of Raw cells stimulated with CSE. Immunofluorescence and flow cytometry were used to detect the polarity of macrophages in the condition of glutamine abundance and deficiency. Cell Counting Kit-8 (CCK-8), wound-healing assay, and Annexin V-FITC (fluorescein isothiocyanate)/PI (propidium iodide) double-staining flow cytometry were applied to detect the growth and transferability and apoptosis of Leuk-1 cells in the supernatant of Raw cells which were stimulated with CSE, glutamine abundance and deficiency. Hyperkeratosis and dysplasia of the epithelium were evident in smoking mice. M2 macrophages increased under CSE stimulation in vivo and in vitro. In total, 162 types of metabolites were detected in the CSE group. The metabolites of nicotine, glutamate, arachidic acid, and arginine changed significantly. The significant enrichment pathways were also selected, including nicotine addiction, glutamine and glutamate metabolism, and arginine biosynthesis. The results also showed that the supernatant of Raw cells stimulated by CSE could induce excessive proliferation of Leuk-1 and inhibit apoptosis. Glutamine abundance can facilitate this process. Cigarette smoke promotes oral leukoplakia via regulating glutamine metabolism and macrophage M2 polarization.

## Introduction

Smoking is a key risk factor for cancer, chronic obstructive pulmonary disease, and cardiovascular disease. Cigarette smoke produced by smoking contains about 4 000 compounds, most of which are harmful to humans, including carcinogens, mutagenic substances, and immunotoxin substances, which have detrimental effects on systemic and local immunity.^1^ Oral mucosa and gingival epithelium are the first human tissues exposed to cigarette smoke and are directly affected by smoking. Smoking can promote the development of various oral diseases, such as oral leukoplakia (OLK), oral cancer, and periodontitis.^2,3^

Smoking is one of the essential epidemiological risk factors recognized by oral leukoplakia, and 80%–90% of OLK patients smoke.^4^ Smoking increases the risk of oral leukoplakia and promotes cancer formation, causing the occurrence of oral squamous cell carcinoma.^5^ The prevalence of OLK is related to the number and duration of smoking, and there is a dose–effect relationship.^6^ The smoking period, the number of cigarettes smoked per day, and the smoking manner all have an impact on the risk of OLK.^7^

However, the mechanism by which smoking promotes OLK pathogenesis is unclear. Biochemically and pathologically, there is strong evidence for airway sensitization, hyperresponsiveness, and inflammation as a consequence of exposure to smoke particulate.^8^ Smoking is recognized to promote oral and respiratory infections caused by biological pathogens. The influence of smoking on immunity is well recognized.^9,10^ Smoking impacts both innate and adaptive immunity and plays dual roles in regulating immunity by either exacerbation of pathogenic immune responses or attenuation of defensive immunity.^11^ Pappas examined studies some of which implied activation of innate response in animals with exposure to smoke, and some of which implied suppression of innate response.^8^ It was noted that innate response was indeed activated with low initial exposures to either cigarette smoke or diesel exhaust particulate, but that at high doses, the innate response was suppressed. Macrophages perform a variety of functions like inflammatory and antimicrobial activity in host defense, resolution of inflammation and wound healing, and maintenance of various homeostatic processes.^12^ It is suggested that the effects of smoking on innate immunity are related to the dose and duration of action. It incorporates the activation of M1 innate response at a low dose but suppresses the M1 innate response at high-dose particle exposures.

Abundant studies have shown that macrophage polarization depends on the environment of human blood monocytes and mouse macrophages in vitro.^13,14^ Smoke stimulation affects the immune environment by affecting the polarization and phagocytosis of macrophages. Research on the effects of smoking on macrophages has been ongoing. Two main macrophage activation programs are called M1 and M2 polarization.^15^ Data indicate that smoking does change the steady-state polarization program in human alveolar macrophages. There is an overall anti-inflammatory gene expression pattern in the macrophage of healthy smokers. The inactivation of the M1 polarization pattern is accompanied by the induction of abnormal phenotypes, characterized by the upregulation of genes associated with different M2 polarization programs. Long-term smoking reprograms the steady-state macrophage polarization to M1 inactivated, and partially M2-activated macrophages, which enhances the ability of tissue remodeling but reduces the gene expression related to inflammation and immunity.^16,17^ Recently, many studies have focused on the metabolic changes of immune cells, and external stimuli are believed to reprogram the metabolism of immune cells, thereby affecting their immune function and differentiation, and subsequently producing many effects.^18^ Research showed that different metabolic pathways are used to regulate macrophage phenotypes. Its functional plasticity and macrophage polarization are related to metabolism.^19^

Metabolic processes and their products play a critical regulatory role in the polarization of mature macrophages, and this local microenvironment and cells are often considered as interaction. Activation or polarization of macrophages by microenvironmental cues can trigger distinct changes in their metabolic program. The cross-talk between the intracellular signal cascades, metabolic pathways, and their metabolites in turn affect the transcription and epigenetic events, resulting in distinct functional states.^20^ The manipulation of such metabolic pathways in these cells can dramatically alter their specific immune functions, rather than simply affecting energy generation or general biosynthesis. Different intracellular metabolic pathways regulate the polarization and function of activated macrophages.

Activation of macrophages to an M1 state by inflammatory stimuli, like lipopolysaccharide (LPS) and interferon-γ (IFNγ), was associated with enhanced glycolysis and impaired tricarboxylic acid (TCA) cycle and mitochondrial oxidative phosphorylation (OXPHOS).

Several recent studies demonstrate an integral role of glutamine metabolism in M2 macrophages, rather than M1 macrophages. Jha et al.^21^ showed glutamine metabolism as a characteristic feature of M2 macrophages.

In recent years, macrophage metabolic reprogramming has attracted attention.^22^ However, how macrophage metabolism is regulated by the local microenvironment stimulated by cigarettes especially for oral mucosa disease is still minimal which is worthy of further study.

In this study, the effect of smoking on macrophage polarization was verified in vitro and in vivo studies. The liquid chromatography-mass spectrometry (LC-MS) non-targeted metabolome analysis was used to screen the significantly different metabolites and enriched metabolic pathways of macrophage by CSE stimulation. We want to verify and explain that if cigarette smoke induced glutamine metabolism and macrophage M2 polarization to affect epithelial hyperplasia and influence the occurrence and development of oral leukoplakia.

## Results

### Smoking promotes oral leukoplakia (OLK) of mice

**Fig. 1:**
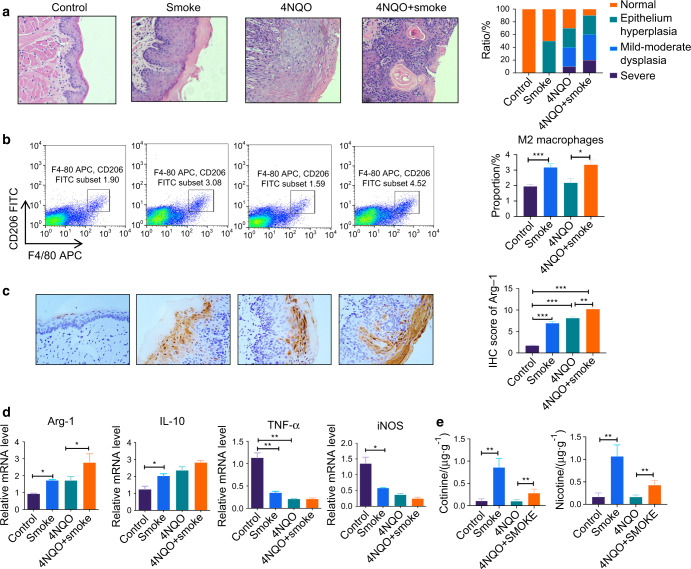
The cigarette smoke promotes OLK development in the C57BL/6 J mice. The mice were fed water with 4NQO and got cigarette smoke twice a day. All Mice were sacrificed at week 16. **a** shows histological analysis of oral mucosa epithelial in four groups with hematoxylin and eosin staining. **b** shows a representative image of the frequency of M2 macrophages (F4/80^+^CD206^+^ cells) from tongue tissue. **c** shows immunohistochemical analysis of Arg-1 in the oral mucosa epithelial tissue of the mice from the four groups. **d** Shows the relative mRNA level of *Arg-1, IL-10, iNOS*, and *TNF*-α in the tongue tissue of the mice. **e** shows nicotine and cotinine contents in mice hair of the four groups. Error bars, SEM. *P < 0.05, **P < 0.01, ***P < 0.001

The mice were given smoking and/or 4NQO (4-nitroquinoline N-oxide), respectively. It was found that after 16 weeks of oral smoking, white streaks or plaques appeared on the tongue of the mice, and tumors were developed in some mice. Most mice in the 4NQO-drinking group showed obvious markings or even masses in the tongue tissues of which pathological examination showed epithelial hyperkeratosis and partial epithelial dysplasia. Meanwhile, the combination of smoke and 4NQO led to a higher proportion of abnormal tongue tissues, compared with a single treatment. Tongue tissues were further analyzed using hematoxylin and eosin (H&E) staining (Fig. 1a). Compared with the control group, leukoplakia and epithelial keratosis were significantly increased in the smoke group. The epithelium hyperplasia of the oral mucosal epithelium was severe in the 4NQO + smoke group, compared with the single-treatment group. While nicotine and cotinine contents assessed by high-performance liquid chromatography (HPLC) are shown in Fig. 1e. The nicotine and cotinine levels of the smoking mice increased significantly.

### Smoking affects macrophage M2 polarization

In the above animal model, the proportion of M2 macrophages (F4/80^+^CD206^+^ cells) was significantly higher in the smoking group than that in the non-smoking group (Fig. 1b). mRNA expressions of genes associated with M2 macrophage function (*Arg-1*, *IL-10*) were also increased in the smoking group compared with the control group. Meanwhile, mRNA expressions of genes associated with M2 macrophage function (*iNOS* and *TNF*-α) were significantly decreased in the smoking group (Fig. 1d). Immunohistochemical analysis of Arg-1 in the oral mucosa epithelial tissue of the mice is shown in Fig. 1c.

**Fig. 2:**
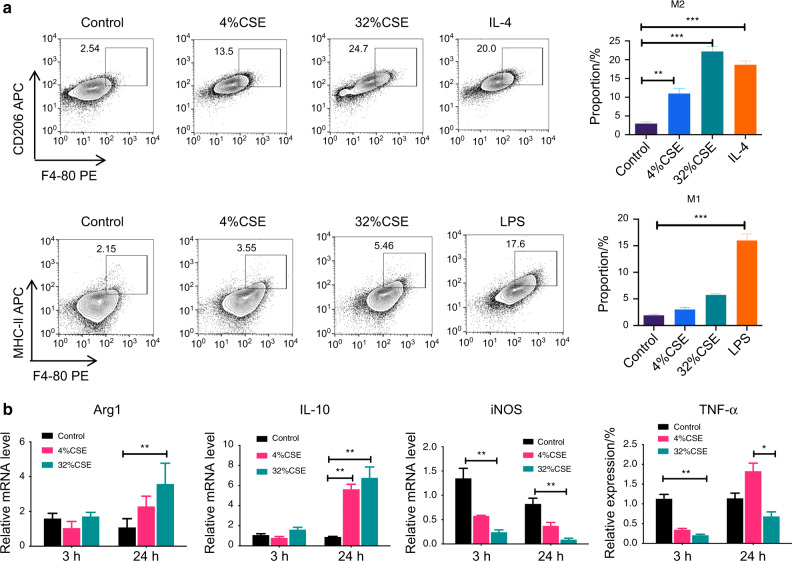
Raw cells stimulated by CSE showed significant M2 polarization. **a** shows a representative image of the frequency of M1 macrophages (F4/80^+^MHC-II^+^ cells) and M2 macrophages (F4/80^+^CD206^+^ cells). **b** shows cytokine expression levels in macrophages were assayed using qPCR. Data represent one of two independent experiments. Error bars, SEM. *P < 0.05, **P < 0.01, ***P < 0.001

Raw cells were stimulated with CSE in vitro. Compared with the control group, the proportion of M2 macrophages (F4/80^+^CD206^+^ cells) in the smoking group was significantly increased, while the proportion of M1 macrophages (F4/80^+^MHC-II^+^ cells) was decreased (Fig. 2a). Similarly, the mRNA levels of *Arg-1* and *IL-10* were increased, while the mRNA levels of *iNOS* and *TNF-a* were decreased in CSE-treated Raw cells (Fig. 2b).

### Smoking promotes glutamine metabolism of macrophages

**Fig. 3:**
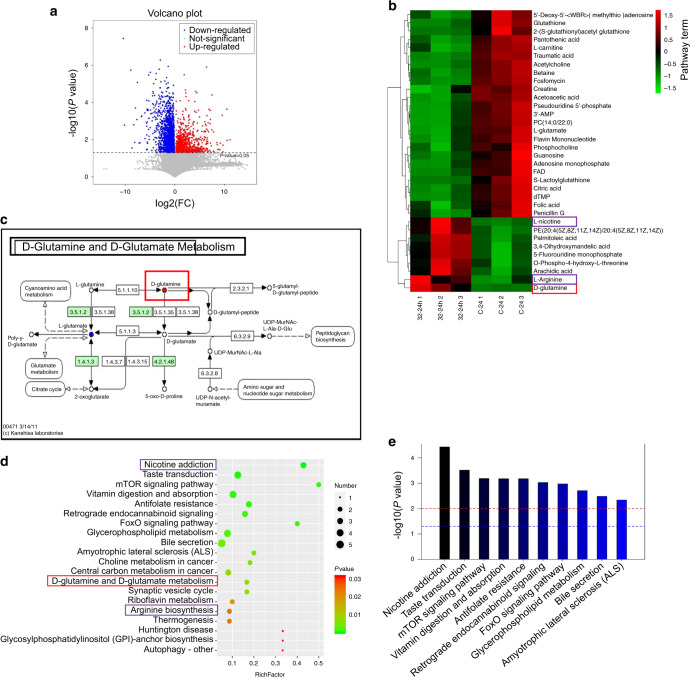
Metabolites and the enrichment pathways of the CSE group compared to the controls. In the volcanic plots, red, blue, and gray dots, respectively, represent upregulated, downregulated, and nonsignificant differential metabolites. Comparing the CSE group with the control group, 162 differential metabolites were detected by LC-MS (*P* < 0.05) (**a**). **c** Shows the d-glutamine and d-glutamate metabolism. Heatmaps of metabolites that can be enriched into known metabolic pathways (**b**). The significant enrichment pathway is selected for bubble mapping (**d**) and a histogram (**e**). The *P* value in the metabolic pathway is the significance of enrichment of this metabolic pathway. The abscissa is the enrichment factor (Rich factor, Rich factor = significantly different number of metabolites/total number of metabolites in the pathway). The greater the Rich factor is, the greater the enrichment degree will be. The color from red to green indicates that the *P* value decreases successively. The larger the dot is, the more metabolites are enriched in this pathway. In panel **e**, the red line indicates the *P* value of 0.01, and the blue line indicates the *P* value of 0.05. When the top of the bar is higher than the blue line, the signal pathway represented by it is significant

The results of non-targeted ultra-high-performance liquid chromatography-mass spectrometry (UPLC-MS), including differential metabolites and enriched pathway terms are shown in Fig. 3. The cells were cultured with 32% CSE for 24 h or Dulbecco’s modified eagle medium (DMEM) (the control group).

Comparing the CSE group with the control group, 162 differential metabolites were detected by UPLC-MS (*P* < 0.05) (Fig. 3a). In the volcanic plots, red, blue, and gray dots, respectively, represent upregulated, downregulated, and nonsignificant differential metabolites.

Heatmaps of metabolites that can be enriched into known metabolic pathways (Fig. 3b). In the CSE group, we found metabolic increased in some organic heterocyclic compounds, such as l-nicotine, d-glutamate, arachidic acid, and l-arginine.

According to the Kyoto Encyclopedia of Genes and Genomes (KEGG) and UPLC-MS data, the significant enrichment pathway is selected for bubble mapping and a histogram (Fig. 3c-e). The *P* value in the metabolic pathway is the significance of enrichment of this metabolic pathway. The abscissa is the enrichment factor (Rich factor, Rich factor = significantly different number of metabolites/total number of metabolites in the pathway). The greater the Rich factor is, the greater the enrichment degree will be. The color from red to green indicates that the *P* value decreases successively. The larger the dot is, the more metabolites are enriched in this pathway. The enrichment analysis of different metabolites is helpful to understand the mechanism of metabolic pathways in different samples.

In the CSE group, the pathways of nicotine addiction, mammalian target of rapamycin (mTOR) signaling pathway, antifolate resistance, glycerophospholipid metabolism, choline metabolism in cancer, central carbon metabolism in cancer, d-glutamine and d-glutamate metabolism, synaptic vesicle cycle, and arginine biosynthesis significantly enriched (Fig. 3d, e).

**Fig. 4:**
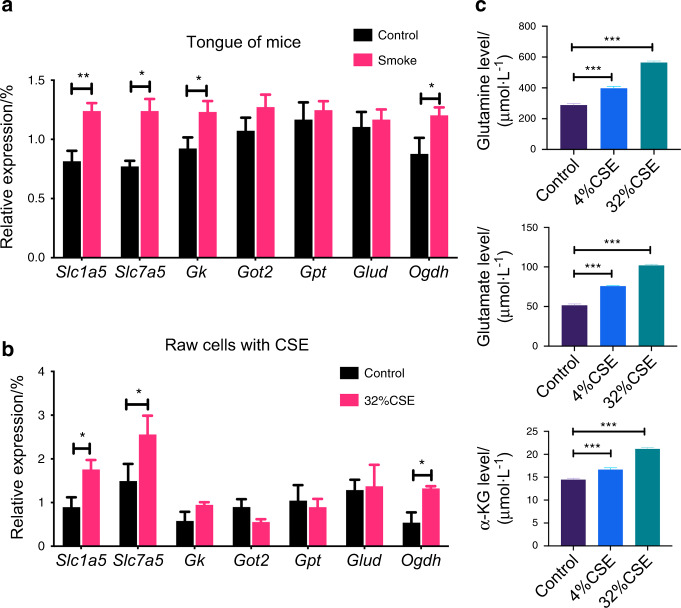
The expression of glutamine after stimulating of cigarette smoke. Glutamine-related gene expression in tongue tissues and RAW cells after CSE stimulation are shown in panels **a** and **b**. In the CSE stimulation group, the relative expressions of genes *Slc1a5*, *Slc7a5*, and *Ogdh* were significantly increased. **c** shows that the expression of glutamine, glutamate, and *α*-KG in the Raw cells under CSE stimulation were significantly increased. Error bars, SEM. *P < 0.05, **P < 0.01, ***P < 0.001

We further verified that in the animal model, the relative expression of *Slc1a5*, *Slc7a5*, and *Ogdh* in the oral mucosa tissue of the smoking group was significantly increased (Fig. 4a). Similar results of the relative expression of *Slc1a5*, *Slc7a5*, and *Ogdh* were found in Raw cells stimulated with CSE (Fig. 4b). The glutamine, glutamate, and α-ketoglutarate (α-KG) contents inside macrophages stimulated by CSE were increased (Fig. 4c).

### Glutamine metabolism affects macrophage M2 polarization

**Fig. 5:**
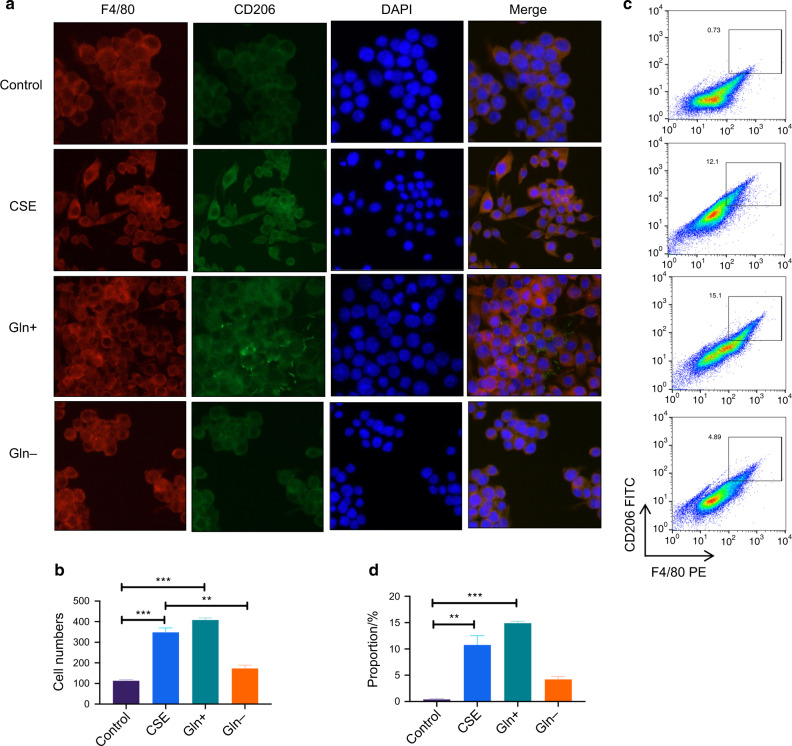
F4/80^+^CD206^+^ Raw cells were significantly increased after CSE stimulation and Glnglutamate abundance. **a**, **b** show the immunofluorescence of F4/80^+^CD206^+^ cells after CES stimulation or under glutamate abundance(Gln+) and deprivation(Gln−) environment. **c**, **d** show the representative image of frequency of F4/80^+^CD206^+^. Error bars, SEM. **P < 0.01, ***P < 0.001

To investigate the role of glutamine metabolism in smoking-promoting macrophage polarization, we added glutamate in the DMEM for the glutamate abundance environment and used the DMEM culture without glutamate for the deprivation environment. Figure 5a, b shows the expression of F4/80^+^CD206^+^ cells by immunofluorescence. Figure 5c, d shows the proportions of F4/80^+^ CD206^+^ cells by flow cytometry. The results showed that F4/80^+^CD206^+^ cells were significantly increased after CSE stimulation and glutamate abundance.

### Glutamine metabolism promotes epithelial cell proliferation

**Fig. 6:**
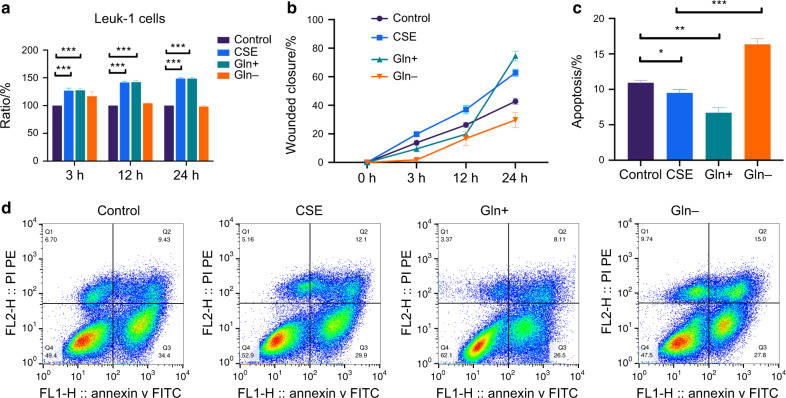
Raw cells were stimulated with or without CSE for 24 h. The supernatant of Raw cells taking four kinds of stimulations (control, CSE, glutamine abundance, and deprivation) was collected. The Leuk-1 cells were cultured in the supernatant of Raw. CCK-8 results are shown in **a**. Migration ability of Leuk-1 cells were analyzed using a wound-healing assay in the presence of Raw cells supernatant (**b**, Fig. S1). The apoptosis ratios are shown in panels **c** and **d** which were detected by flow cytometry with Annexin V- FITC/PI double staining. Error bars, SEM. *P < 0.05, **P < 0.01, ***P < 0.001

In order to explore the role of glutamine metabolism in promoting macrophage M2 polarization and inducing epithelial cell proliferation by cigarette smoking, we performed a wound-healing assay to examine the migration ability of Leuk-1 cells under the supernatant of Raw cells cultured with or without glutamate. It was found that cells with the Raw cell supernatant stimulated with CSE and glutamate were more active, while the supernatant of Raw cells using glutamine-free medium was significantly less effective (Fig. 6b and S1). CCK-8 results are shown in Fig. 6a and the apoptosis ratios are shown in Fig. 6c, d which were detected by flow cytometry with Annexin V-FITC/PI double staining. The abundance of glutamine significantly induced Leuk-1 cell viability and inhibited apoptosis rate in Leuk-1 cells compared with treatment with CSE alone.

## Discussion

Smoking, as an important risk factor of OLK, has long been one of the concerns in the research field of the oral mucosa. There are many studies on the direct effects of smoke stimulation on epithelial cells. However, few studies are on the effects of smoke stimulation on the differentiation and function of oral local immune cells. In this study, the results of animal models and cell experiments in vitro showed that smoke promoted the differentiation and M2 polarization of macrophages in oral mucosa to affect the dysplasia of epithelial cells indirectly.

Macrophages, the mature form of the monocytes, play a significant role in tissue homeostasis and immunity. Macrophages are an important cell type that play a wide variety of roles in various organ sites. Cigarette smoking is a major pathogenic factor in lung cancer. Macrophages play an important role in host defense and adaptive immunity. These cells display diverse phenotypes for performing different functions. Exposure to particulate induces both M1 and M2 polarization. The results of this research show M2 polarization is increased with exposure to smoke, while the dose is the important consideration as mentioned in Pappa’s review.^8^ As M1 macrophages undergo M2 polarization, fewer M1 macrophages are available. As fewer M1 macrophages produce M1 proinflammatory cytokines, fewer monocytes are recruited from bone marrow to replace them, so polarization is shifted to M2. It is not mean that M2 polarization suppresses M1 immune response nor the M1 tumor-suppressing activity. M2 polarization is still an immune response in which the T2 activity of T cells promotes antibody production. Smokers often have higher circulating IgE than nonsmokers and light smokers, indicating that sensitization is active, but often the atopic response associated with high IgE levels is suppressed as well. While at high particle dose exposures, M2 immune response is also suppressed. Therefore, it is reasonable to expect that cigarette smoke promotes a M2-like phenotype with dose dependence.^23^

These effects are mediated through a few major pathways including the nuclear factor kappa-B (NF-κB), mitogen-activated protein kinase (MAPK), and Janus kinase and signal transducer and activator of transcription (JAK/STAT) signaling pathways.^5,24^ It is showed that in our study, the metabolism of macrophages changes significantly when it gets the smoke stimulation. This suggests that metabolic reprogramming may be one of the mechanisms for smoking to promote OLK.

In this experiment, we performed two sample groups for the metabolite detection by LC-MS, and metabolic effects of CSE stimulation on Raw cells were studied. Liquid chromatography-mass spectrometry (LC-MS) has distinct advantages over other metabolic grouping techniques.^25^ Ultra-high-pressure liquid chromatography or ultra-high-performance liquid chromatography (UPLC) achieves better separation. In MS scanning, the tandem mass spectrometer can achieve rapid switching of high- and low-collision energy. Combined with mass spectrometry information analyzed by Progenesis QI v2.3, abundant metabolites can be detected.^26^

After CES stimulation, Raw cells had nicotine, cocaine, and morphine substance dependence, and multiple metabolites changed in nicotine, cocaine, and morphine addiction, which were related to metabolite changes such as acetylcholine and glutamate. In the ventral tegmental area (VTA), alpha6-nicotinic acetylcholine receptors and alpha4beta2-nicotinic acetylcholine receptors (α6nAChRs and α4β2nAChRs) inhibit DAergic neurons, while alpha7-nicotinic acetylcholine receptors (α7nAChRs) enhance glutamate release and increases the excitability of DAergic neurons. After short-term nicotine exposure, α6nAChRs and α4β2nAChRs on GABAergic terminals are desensitized, reducing γ-aminobutyric acid (GABA) release and local inhibition of dopamine (DA) neurons. The α7nAChRs on the glutamate terminal remain active and enhance the glutamate excitation of DA neurons and DA release. After literature review, it is known that α7nAChR is expressed in various nonneuronal cells, including macrophages.^27,28^ This may be one of the reasons for the abnormal glutamine metabolism in macrophages induced by smoke stimulation.

The result of LC-MS showed that in amino acid metabolism, glutamine rose significantly in the CES group. Glutamine metabolic pathways in the smoke group enriched significantly, while the M2 macrophage polarization happened significantly. It was pointed that when macrophage got smoke stimulation, glutamine metabolic and M2 polarization played important roles. Early studies have indicated a role for glutamine in LPS-stimulated macrophages and the expression of cytotoxic/inflammatory effectors such as NO and IL-1β. In LPS-activated M1 macrophages, glutamine metabolism contributes to the increased generation of succinate via anaplerosis (proceeding through α-ketoglutarate α-KG) and γ-aminobutyric acid (GABA) shunt.^29^ However, several recent studies demonstrated an integral role of glutamine metabolism in M2 macrophages, rather than M1 macrophages.^30,31^ Glutamine metabolism acted as a characteristic feature of M2 macrophages. They showed that almost a third of the carbons in the TCA cycle were derived from glutamine.^21^ Functionally, deprivation of glutamine or inhibition N-glycosylation decreased the expression of several M2 markers like Il4i1 and CD206.

Jha et al. characterized systemic changes during murine macrophage M1 and M2 polarization. Glutamine deprivation affects M2 polarization but not M1 polarization.^21^ Glutamine deprivation shows a significant (50%) defect in M2 commitment in glutamine-deprived media based on CD301-CD206. M2 polarization was found to activate glutamine catabolism and UDP-GlcNAc-associated modules. Correspondingly, glutamine deprivation or inhibition of N-glycosylation decreased M2 polarization.

At the same time, significant increases in arginine content were found in both arginine and proline metabolism and arginine biosynthesis pathways. l-Arg in macrophages was regulated by iNOS and Arg-1, and iNOS can catalyze l-Arg to produce NO and l-citrulline. NO had a bactericidal effect of meeting the M1 macrophage needs for anti-inflammatory and pathogen invasion resistance, and l-citrulline was used in the urea cycle.^32^ Inversely, Arg-1 catalyzed l-Arg to produce ornithine and urea ammonia acid, promoting cell proliferation and collagen synthesis and working for tissue repair and remodeling. High expression of Arg-1 was detected in M2 macrophages.^33^ The qPCR and flow cytometry results showed that M2 macrophages significantly increased especially under CSE stimulation, suggesting that amino acid metabolism and product changes are involved in the smoke-stimulation mechanism.

We found common changes in glutamate, arginine, and citric acid in the arginine-proline synthesis pathway. metabolic changes in three glutamate derivatives were found in the glutamine metabolic pathway. l-glutamate decreased and d-glutamine increased in the d-glutamine and d-glutamic acid metabolism. The expression of glutamic acid and arginine changed under 32%-24 h CSE stimulation. The significant increase in arginine metabolism in the stimulation suggested tissue repairment.

The mechanism of glutamine metabolism in the process of macrophage M2 polarization induced by smoking is worth exploring. Glutamine metabolism is recognized to be associated with tumors. However, the role of glutamine metabolism in smoking promoting the precancerous lesions of oral mucosa is still unknown.

In this study, the result showed the expression of *Slc1a5* and *Slc7a5* genes increased significantly in the CSE group. The uptake of glutamine through the cytomembrane mainly relies on translocators, such as ASCT2 (system ASC amino acid array 2) and LAT1(l-type amino acid array 1).^34,35^

*Slc1a5* and *Slc7a5* are transcription genes of ASCT2 and LAT1. Microarray Data raised to elevated expression of ASCT2 (*Slc1a5*) and LAT1 (*Slc7a5*) in many cases, and this has been confirmed in many studies and cell lines.^36,37^ ASCT2 high expression generally predicts a poor prognosis in cancer patients.^38–40^

According to this study, smoke activates the glutamine translocators in macrophages, promotes the intracellular transport of glutamine, leads to the active metabolism of glutamine, changes the local immune metabolism microenvironment of the oral mucosa, and promotes abnormal cell proliferation and reduces cell apoptosis. The effects of glutamine abundance and deficiency on macrophages and epithelial cells in this study further confirm this inference.

Therefore, it is concluded that in the process of smoke cigarettes promoting OLK, glutamine metabolism and M2 polarization of macrophage play important roles for regulating oral mucosa immune microenvironment and epithelial dysplasia and keratosis. Still, the specific mechanism involved may need more experimental data to be fully depicted.

## Materials and methods

### Animal studies

All animal studies were approved by the Animal Ethical and Welfare Committee of Nanjing University (IACUC-2003136). Seven-week-old C57BL/6J male mice purchased from Qinglongshan, Inc., Nanjing, China, received regular food and water before the experiment. 4NQO powder was dissolved in ultra-pure water, the concentration is 50 μg·mL^−1^. All mice were divided into two groups. An OLK model group drunk 4NQO-dissolved water as daily drinking water for 16 weeks and a negative control group drunk water. Half of them received air exposure and others exposed to cigarette smoke. There were four groups: (I) the control group; (II) the smoke group; (III) the 4NQO group; and (IV) the 4NQO and smoke group (*n* = 10).

Smoke was prepared, as previously described.^41^ Mice in the smoke group smoked two cigarettes every day. All the mice were sacrificed after 16 weeks to get oral tissues. Some samples were stored at −80 °C for qPCR and flow cytometry, and others were embedded in paraffin for histological analysis by H&E stain. Histological scores were assessed by a pathologist. Nicotine and cotinine contents in mouse hair were detected by HPLC.

### Cell lines and cell cultures

Raw cells were obtained from the Shanghai Institute of Cell Biology (Shanghai, China) and cultured with DMEM basic (1×) (Gibco, Thermo Fisher Scientific) at 37 °C in 5% CO_2_ and humidified air incubators.

Leuk-1 cells, an immortalized human oral mucosal epithelial cell line, were a generous gift from Professor Li Mao in the Department of Oncology and Diagnostic Sciences, University of Maryland Dental School, Baltimore, MD, USA. Leuk-1 cells were cultured and passaged in a defined keratinocyte serum-free medium (K-SFM) (Gibco, Invitrogen, Carlsbad, CA, USA).

### CSE preparation

3R4F Kentucky reference cigarettes were purchased from the Tobacco Research Institute at the University of Kentucky (Lexington, KY, USA). CSE was prepared by a peristaltic pump where one 3R4F reference cigarette smoke was bubbled through 10 mL of keratinocyte serum-free medium. Then the solution was filtered, diluted, and administered to cell cultures within 30 min.

### Metabolomic analysis

#### Sample preparation

Raw cells were supplemented with CSE (4%/32%). Samples were collected 3 h and 24 h later. We discarded the medium, added internal standard (2-chlor-1-phenylalanine, 0.3 mg·mL^−1^) and 1 mL methanol: water (V:V = 4:1), and transformed samples to a 4-mL glass vial where 200 μL trichloroethane were added. Sample extracts were centrifuged at 13 000 r·min^−1^, 4 °C for 10 min. In total, 400 μL methanol: water was added. Samples centrifuged, the supernatants (150 μL) were collected, filtered, and transferred to LC vials stored at −80 °C until metabolomic analysis.

#### UPLC-MS analysis

The metabolomics analysis was conducted using UPLC-MS in ACQUITY I-Class system and VION Ion Mobility Spectrum Quadrupole Time-of-Flight mass spectrometer in both positive and negative modes (Waters Corporation, Milford, MA). Water and acetonitrile/methanol (V:V = 2:3) both containing 0.1% formic acid were used as mobile phases A and B. Linear gradient: 0 min, 1% B; 1 min, 30% B; 2.5 min, 60% B; 6.5 min, 90% B; 8.5 min, 100% B; 10.7 min, 100% B; 10.8 min, 1% B and 13 min, 1% B. The flow rate was 0.4 mL·min^−^^1^, and the column temperature was 45 °C.

We alternatively acquired data with full scan mode (m/z ranges from 50 to 1 000), which is combined with a mode including two independent scans with different collision energies (CE). A low-energy scan (CE 4 eV), and a high-energy scan (CE ranp 20–45 eV) were the parameters of mass spectrometry.

Argon (99.999%) was used as collision-induced dissociation gas; scan rate: 0.2 s/scan; capillary voltage, 1 kV (negative mode)/2 kV (positive mode); reference capillary voltage, 2.5 kV; cone voltage, 40 V; source offset, 60 V; source temperature, 115 °C; desolvation gas temperature, 450 °C; desolvation gas flow, 900 L·h^−1^, and cone gas flow, 50 L·h^−1^ nitrogen (>99.5%) was employed as desolvation and cone gas. For lock mass correction, a 250 ng·mL^−1^ standard solution of leucine-enkephalin in acetonitrile/water/formic acid (50: 49.9: 0.1, v/v/v) was continuously infused (5 μL·min^−1^) through the reference probe and scanned every 30 s.

#### Data preprocessing and statistical analysis

The raw data acquired from LC-MS were analyzed by the progenesis QI software (Waters Corporation, Milford, USA). Supervised orthogonal partial least squares discriminant analysis (OPLS-DA) was performed to visualize the alterations of metabolites between the groups.

Metabolites were identified by the progenesis QI software (Waters Corporation, Milford, USA) based on the Human Metabolome Database (HMDB, http://www.hmdb.ca/), LIPID MAPS database (http://www.lipidmaps.org/) and the self-built database of Shanghai Lu-Ming Biotech Co., Ltd (Shanghai, China). The differential metabolites were screened by the combination of multidimensional analysis and unidimensional analysis. The thresholds were set to variable important for the projection (VIP) obtained from the OPLS-DA > 1 and *P* value from a two-tailed Student’s test <0.05.

In order to identify the effect of disturbed metabolites on metabolic pathways, pathway enrichment analysis for differential metabolites was performed using MBRole 2.0 (http://csbg.cnb.csic.es/mbrole2/) based on Kyoto Encyclopedia of Genes and Genomes (KEGG, http://www.genome.jp/KEGG/pathway.html). The pathway with *P* value <0.05 was identified as the significant pathway.

### RT-qPCR

Total RNAs of cells and tissues were extracted with Trizol Reagent (Invitrogen, Carlsbad, CA) and were reverse-transcribed into cDNA using oligo (dT) primer. Step One Plus or an ABI Vii 7 detection system (Applied Biosystems, Thermo Fisher Scientific, US) with SYBR Green PCR master mix solution was used for qPCR. The primers used are listed in Supplemental Table 1.

### Flow cytometry

Raw cells were washed and stained with fluorescent-conjugated antibodies. Anti-mouse antibodies (Biolegend, San Diego, CA, US) were used: anti-F4/80 PE (#123110), anti-MHC-II APC (#107613), and anti-CD206 APC (#141708). FACS Calibur flow cytometer (Becton Dickinson, Franklin Lakes, NJ) detected cells. FlowJo software (Treestar, Inc., San Carlos, CA) analyzed the data.

### Immunofluorescence

Cell samples in six-well plates were fixed with acetone for 15 min after different treatments. Then, the samples were permeabilized with 0.3% Triton X-100 for 20 min, washed with PBS, and blocked with PBS containing 3% bovine albumin (BSA) at 37 °C for 30 min. Subsequently, primary antibodies against CD206 and MHC II (diluted 1:1 000 in 3% BSA) were added and incubated at 4 °C overnight, and goat anti-rabbit FITC 488 (1:1 000; CST) and CY3 554 (1:1 000; CST) were placed onto the coverslips and incubated at 37 °C for 30 min. After washing in PBS, the cellular nuclei of each sample were counterstained with diamidino phenylindole (DAPI). After staining, the samples were observed using a confocal fluorescence microscope (FV1000, Olympus, Tokyo, Japan).

### Measurement of cell viability

Cell viability was measured by the Cell Counting Kit-8 (CCK-8) (Dojindo Laboratories, Kumamoto, Japan) assay. Leuk-1 cells at a density of 3 000 cells per well were cultured in 96-well plates at 37 °C and 5% CO_2_. Each well was treated with supernatant from Raw cells under different stimulations (DMEM, DMEM + CSE, DMEM + 4 mmol·L^−1^ Gln, DMEM without Gln), for different lengths of time (3 h, 12 h, 24 h) after cell adhesion. Then, the supernatants were discarded, and 10 μL of CCK-8 solution was added to each well. After 2 h of incubation, the absorbance at a wavelength of 450 nm was determined by using a multiplate reader (BioTek, CA, USA).

### Wound-healing assay

The Raw cell supernatants stimulated by CSE (32%, 24 h) were collected. The initial wound size of Leuk-1 cells was determined immediately after washing the cells. The supernatants of Raw cells were added to the medium of Leuk-1 cells, including four groups (Control, CSE, Gln+ , Gln-). After 3, 12, and 24 h, the wound closure was calculated as the percentage of the remaining wound area.

### Glutamine colorimetric assay

To prepare the samples for the glutamine colorimetric assay kit (Abcam), tissue samples were washed in 1× PBS and re-suspended in hydrolysis buffer on ice. Then, tissues were homogenized using a homogenizer with about 15 passes, then centrifuged at 10 000 × *g*, 4 °C for 10 min. Deproteinization was performed on the supernatant, with the addition of ice-cold 4 mol·L^−1^ PCA to a final concentration of 1 mol·L^−1^. Samples were vortexed briefly and incubated on ice for 5 min, then centrifuged at 13 000 × *g*, 4 °C for 2 min. An equal volume of 2 mol·L^−^^1^ KOH was added to the supernatant and vortexed to adjust the pH to 6.5-8. Samples were centrifuged at 13 000 × *g*, 4 °C for 5 min. The supernatant from samples was transferred to newly, labeled tubes for glutamine assay. Briefly, 40 mL of glutamine standard and diluted samples were added to a 96-well plate. In all, 2 mL of hydrolysis mix was added to glutamine standards and sample wells and incubated for 30 min at 37 °C 50 ml of glutamine reaction mix was added to wells and incubated for 60 min at 37 °C. Absorbance was measured at OD-450 nm on a microplate reader (Synergy HT, BioTek).

### Statistical analysis

All the experiment data of biochemical index were presented as means ± standard error of the mean (SEM). Statistical analysis of the experimental data was performed by Prism 8 (GraphPad Software). A two-tailed Student’s *t* test and paired *t* test were used. The significance threshold and extremely significance threshold were set at *P* value < 0.05 (*), *P* value < 0.01 (**), and *P* value < 0.001 (***), respectively.

## Supplementary information

Supplementary Table

Fig.S1

## Data Availability

The data that support the findings of this study are available from the corresponding author upon reasonable request.
