# Supplementary material for: Cigarette smoke promotes oral leukoplakia via regulating glutamine metabolism and M2 polarization of macrophage
Source: Int J Oral Sci. 2021 Aug 9;13:25. doi: 10.1038/s41368-021-00128-2 (PMC8352977; doi:10.1038/s41368-021-00128-2)
Supplement: Supplementary file 1 — Supplementary Table [file 41368_2021_128_MOESM1_ESM.docx]

**Tables**

**Supplement Table 1. Primers for qPCR**

| **Gene** | **species** | **Forward (5’→3’)** | **Reverse (5’→3’)** |
| --- | --- | --- | --- |
| SLC1A5 | mouse | CATCAACGACTCTGTTGTAGACC | CTGGATACAGGATTGCGGTATTT |
| SLC7A5 | mouse | ATATCACGCTGCTCAACGGTG | GCCGCCTGACTTGGAGATG |
| GK | mouse | TGAACCTGAGGATTTGTCAGC | CCATGTGGAGTAACGGATTTCG |
| GPT | mouse | TCCAGGCTTCAAGGAATGGAC | GGACTGCATACTCCACCCT |
| GOT2 | mouse | GCGTTACCGAAGCCTTCAAGA | GGAGCACGTAAGGTTTTCCGT |
| GLUD | mouse | CCTGCAACCATGTGTTGAGC | CGGTAGCCTTCGATGACCTC |
| OGDH | mouse | GTTTCTTCAAACGTGGGGTTCT | GCATGATTCCAGGGGTCTCAAA |
| TNF-a | mouse | GCCTCCCTCTCATCAGTTCT | CACTTGGTGGTTTGCTACGA |
| Arg-1 | mouse | CTCCAAGCCAAAGTCCTTAGAG | GGAGCTGTCATTAGGGACATCA |
| iNOS | mouse | GTTCTCAGCCCAACAATACAAGA | GTGGACGGGTCGATGTCAC |
| IL-10 | mouse | CTTACTGACTGGCATGAGGATCA | GCAGCTCTAGGAGCATGTGG |
| β-actin | mouse | GGCTGTATTCCCCTCCATCG | CCAGTTGGTAACAATGCCATGT |
