# Supplementary figures and images for: Cigarette smoke promotes oral leukoplakia via regulating glutamine metabolism and M2 polarization of macrophage
Source: Int J Oral Sci. 2021 Aug 9;13:25. doi: 10.1038/s41368-021-00128-2 (PMC8352977; doi:10.1038/s41368-021-00128-2)

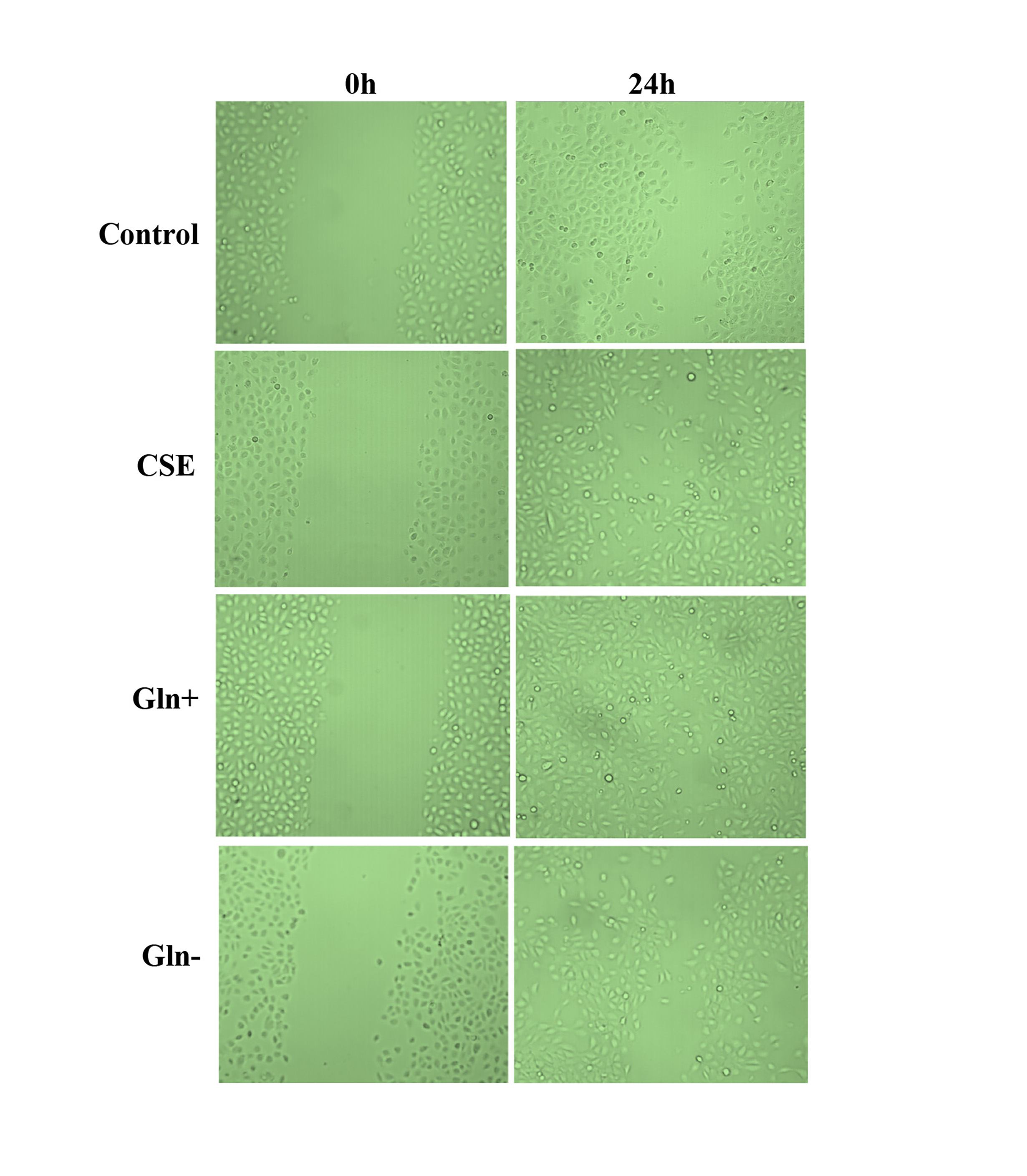

Supplement: Supplementary file 2 — Fig.S1 [file 41368_2021_128_MOESM2_ESM.jpg]
